# Computer vision analysis of luteal color Doppler ultrasonography for early and automated pregnancy diagnosis in *Bos taurus* beef cows

**DOI:** 10.1093/jas/skaf166

**Published:** 2025-05-13

**Authors:** Lucas Melo Gonçalves, Pedro Levy Piza Fontes, Anderson Antonio Carvalho Alves

**Affiliations:** Department of Animal and Dairy Science, University of Georgia, Athens, GA, 30602, USA; Department of Animal and Dairy Science, University of Georgia, Athens, GA, 30602, USA; Institute for Integrative Precision Agriculture – University of Georgia, Athens, GA 30602, USA; Department of Animal and Dairy Science, University of Georgia, Athens, GA, 30602, USA; Institute for Integrative Precision Agriculture – University of Georgia, Athens, GA 30602, USA

**Keywords:** beef cattle, deep learning, fertility, image analysis

## Abstract

This study evaluated the suitability of applying supervised deep learning (DL) algorithms for early and real-time pregnancy diagnosis in beef cattle using luteal color Doppler (CD) ultrasonography recorded on days 20 (D20) and 22 (D22) after fixed-time artificial insemination (FTAI). CD ultrasound videos from 390 females were manually evaluated by trained personnel to perform the human-based pregnancy diagnosis (Human). Images were extracted at a rate of 5 frames per second from each video, resulting in 10,533 (D20) and 10,413 (D22) valid frames after applying a frame-filtering pipeline. Three convolutional neural network (CNN) architectures—VGG19, Xception, and ResNet50—along with their averaged inference (Combined), were evaluated using restricted 5-fold cross-validation, ensuring that images from the same animal did not appear in both training and validation sets. Final inferences for each animal were determined by averaging the network outputs across all video frames. Pregnancy status was confirmed on day 29 using conventional ultrasonography and treated as ground truth for assessing both Human and DL-based predictions. Accuracy levels were similar across methods, ranging from 0.84 (VGG19) to 0.87 (Human) for D20 and from 0.86 (VGG19) to 0.93 (Human) for D22. Based on Matthew’s correlation coefficient, the Combined and Xception architectures demonstrated the best overall agreement with true pregnancy status among DL models. These architectures performed comparably to human diagnosis, with the Combined model achieving similar F1 scores (0.89 vs 0.91), higher specificity (0.72 vs 0.65), and slightly lower sensitivity (0.95 vs 1.00) on D20. Xception showed similar performance to human diagnosis on D22, with comparable accuracy (0.91 vs 0.93), specificity (0.79 vs 0.81), sensitivity (0.99 vs 1.00), and F1 score (0.93 vs 0.94). In conclusion, DL algorithms can effectively predict pregnancy status using CD ultrasonography earlier than industry-standard methods, with performance comparable to that of trained personnel.

## Teaser

Deep learning algorithms applied to luteal Color Doppler ultrasonography accurately classified pregnancy status as early as day 20 after insemination, achieving performance comparable to trained personnel. This approach holds promise for enhancing workflow efficiency in commercial beef cattle reproductive management.

## Introduction

Early pregnancy diagnosis is critical for optimizing reproductive management decisions in beef cattle production systems, allowing producers to improve herd reproduction efficiency by culling infertile cows or rebreeding nonpregnant females, as reviewed by Fontes and Oosthuizen (2022). Rectal palpation can be used for pregnancy diagnosis starting 40 to 60 d after artificial insemination (AI). Improvements in portability, increased autonomy, image quality, and cost reduction of ultrasound (US) technologies for large animal veterinary usage have popularized them as a common pregnancy diagnosis method in commercial settings. Specifically, conventional Brightness mode (B-mode) ultrasonography has been employed for assessing the bovine reproductive tract since the 1980s (Pierson and Ginther, 1984) and currently stands as a highly accurate method for pregnancy diagnosis. However, its efficacy is constrained, allowing accurate diagnoses no earlier than day 28 of gestation (Nation et al., 2003).

Color Doppler ultrasonography (CD) is a relatively newer technology that enables the identification of blood perfusion in specific organs or structures, thereby offering valuable clinical insights into their functionality. Recent studies indicate that greater blood perfusion in the corpus luteum (CL; an ovarian structure responsible for progesterone production and pregnancy maintenance) at the moment of embryo transfer is associated with increased pregnancy rates in cows (Pugliesi et al., 2019; Santos et al., 2023). Additionally, reductions in luteal blood perfusion within the CL in females undergoing luteolysis can be effectively utilized for pregnancy diagnosis before the embryo reaches a size visible through conventional B-mode ultrasonography (Holton et al., 2022a, 2022b). Consequently, CD ultrasonography has been proposed as a novel method to recognize nonpregnant females earlier than B-mode ultrasonography (20 vs 28 d, respectively; Holton et al., 2022 a,b). Nonetheless, processing and interpreting high-quality CD images require highly trained personnel and may exhibit inter- and intra-observer variability. These limitations present a significant challenge for the large-scale application of this technology under commercial conditions.

The use of image processing and analysis techniques could assist veterinarians with the systematic evaluation of CD images, contributing to the automation of complex tasks such as classifying, segmenting, and detecting regions of interest in ultrasound scans. Computer vision systems (CVS) enabled by advanced deep learning (DL) algorithms are one of the driving factors in the recent revolution observed in medical imaging, allowing fast and accurate diagnosis of different diseases (Aggarwal et al., 2021; Anaya-Isaza et al., 2021). Not surprisingly, the interest in CVS for animal and veterinary sciences is rapidly increasing, with numerous successful applications recently reported, including animal identification (Andrew et al., 2021), behavior monitoring (Bresolin et al., 2023), body weight prediction (Cominotte et al., 2020; Xiong et al., 2023), and ultrasound-based disease diagnosis (Dumortier et al., 2022). However, to date, there have been only limited initiatives to develop efficient CVS for systematically processing and analyzing reproductive ultrasound data in cattle (Górna et al., 2017; Andrade et al., 2023). These systems could provide chute-side support to veterinarians by facilitating image interpretation, reducing misdiagnosis, and optimizing the workflow of ultrasound analysis in farm conditions, ultimately enabling more efficient reproductive management decisions in commercial farms. Furthermore, leveraging CVS for analyzing ultrasound data could contribute to the large-scale acquisition of novel phenotypes associated with cattle fertility. This study aimed to evaluate the suitability of using supervised DL algorithms for early and automatic pregnancy diagnosis using luteal CD ultrasonography recorded 20 and 22 d after fixed-time AI.

## Materials and Methods

All procedures performed in this study followed the protocols approved by the University of Georgia’s Animal Care and Use Committee (A2020 02-002-Y3-A0).

### Experimental animals and data acquisition

This study used data from beef cattle (*Bos taurus*) raised in 5 different locations in the states of Georgia, Mississippi, and Virginia. Records from 212 lactating cows (mean ± SD; body weight = 517 ± 69 kg; body condition score = 5.25 ± 0.50; d postpartum = 78.7 ± 19.8) and 178 replacement heifers (body weight = 341 ± 41 kg; body condition score = 5.15 ± 0.50) were obtained from 2 experiments (Holton et al., 2022a,b). For a detailed description of the conducted protocols, please refer to Holton et al. (2022a,b). Briefly, cows and heifers were exposed to the 7-d CO-Synch + CIDR protocol wherein they were administered gonadotropin-releasing hormone (GnRH; 100 μg intramuscularly; Factrel; gonadorelin hydrochloride; Zoetis Animal Health, Parsippany, NJ) and a controlled intravaginal drug release (CIDR) insert (EAZIBREED CIDR; 1.38 g of progesterone; Zoetis Animal Health) on day −10 (Lamb et al., 2006; Larson et al., 2006). Prostaglandin F2α (25 mg intramuscularly; Lutalyse HighCon; dinoprost tromethamine; Zoetis Animal Health) was administered upon CIDR removal on day −3. The animals were then exposed to fixed-time AI (FTAI) protocol and concurrently received a second GnRH injection 60 to 66 h (lactating cows) or 54 ± 2 h (heifers) after CIDR removal.

Brightness mode and CD ultrasonography were performed on D20 and D22 after AI using a Sonoscape S8EXP machine (SonoScape Medical Corp, Shenzhen, GD, China) to evaluate the morphometric traits and blood perfusion of the CL. In total, 396 videos were recorded each day. All videos were recorded at 16 frames per second, with an average length of 10.76 ± 2.37 (sec) for D20 and 10.96 ± 2.36 (sec) for D22. The recorded ultrasound videos were named by animal identification and stored for posterior analysis by trained personnel. The luteal area was measured by the operator using the B-mode tracing function. The two largest diameters of the CL, forming a 90-degree angle, were measured, and the perimeter was outlined manually. Central, peripheral, and total luteal blood perfusion were subjectively estimated as the percentage of the luteal area with blood perfusion signals, following previously described methods (Pugliesi et al., 2014; Holton et al., 2022a,b). The estimates were independently performed by 2 evaluators, and the average of their measurements was used for each luteal response variable. Intra-assay variation coefficients were calculated after the measurements, and any video presenting a difference greater than 10% between the individuals was reevaluated by both individuals, with a new estimate being obtained.

The Human diagnosis was performed under office conditions by 2 highly trained individuals, relying on a combined assessment of luteal morphometry and blood perfusion characteristics (Holton et al., 2022a, b). Pregnancy status was confirmed on day 29 using conventional B-mode ultrasonography, which was considered the gold standard method for pregnancy diagnosis. Cows were only considered pregnant on day 29 if an embryo and uterine fluid, consistent with a day 29 pregnancy, were present in the uterine lumen. The observed pregnancy rates were 56% for cows and 64% for heifers.

### Data preprocessing

**Figure 1.**
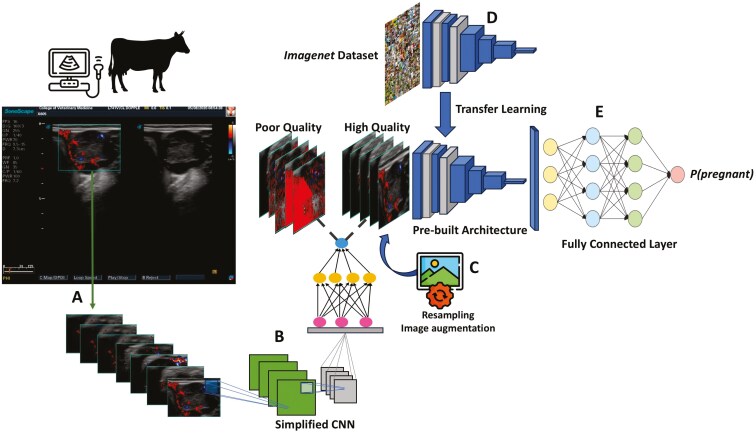
Schematic representation of data acquisition, preprocessing, and analysis steps for classifying pregnancy status in beef cattle using color Doppler ultrasound. Angus cows were scanned with ultrasound on days 20 and 22 after fixed-time artificial insemination. Frames were extracted from recorded videos using bounding boxes marked by the ultrasound software (A). The extracted images were then filtered using a simplified convolutional neural network (CNN) (B). During training, resampling and image annotation were applied (C). Various pre-built architectures, initialized with ImageNet-transferred weights (D), were connected to fully connected layers for classification (E).

Each recorded ultrasound video was used to extract 5 images per second, which were cropped based on the region of interest tracked with the ultrasound software (Figure 1A). This process was automatically implemented using the Python programming language (Van Rossum and The Python development team, 2024) and the OpenCV library (Open Computer Vision Library, 2021). To remove noisy images generated in this process, we randomly labeled 2,289 images from a subset of videos based on their information quality. Images that lacked or had only partial CL presence, as well as those entirely covered by blood perfusion signals due to probe repositioning, were labeled as poor quality (class 0). All other images were considered high-quality frames (class 1). We used this information to train a binary classifier based on a simple convolutional neural network (CNN) architecture.

The CNN model for frame filtering was trained using 1,832 images, while the remaining images were used to monitor its classification accuracy. This model achieved an accuracy of approximately 0.90 in the independent testing set. We used this trained CNN to filter all images in our dataset based on their classified quality (Figure 1B). Each image was considered for further analysis if the CNN inferred a probability >0.95 for class 1. After applying the frame-filtering pipeline in the full dataset, 10,533 (D20) and 10,413 (D22) valid frames were retained, from which around 68% were from pregnant females. These images were then resized to 150 × 150 pixels and rescaled to values between 0 and 1.

During the cross-validation procedure (detailed below), the training images of the positive class (pregnant cows) were randomly down-sampled. This process was performed to address the class imbalance problem, preventing DL algorithms from disproportionately emphasizing the majority class. Subsequently, data augmentation was employed for both classes using Keras’ built-in ImageDataGenerator functionality (Figure 1C). Key parameters were configured as follows: rotation_range = 20, zoom_range = 0.1, horizontal_flip = True, vertical_flip = True, and fill_mode = ‘nearest’. This approach generated novel images during training by introducing random rotations within a 0 to 20-degree range, zoom variations up to 10%, and a 50% probability of horizontal or vertical flips. Our objective was to increase the diversity and robustness of the training datasets, facilitating improved model generalization and performance. It is important to highlight that the sampling and data augmentation techniques described above were not applied to the validation and testing sets, maintaining the class distribution and image settings according to real-world conditions.

### DL algorithms

In this study, we evaluated the performance of three different CNN architectures: VGG19 (Simonyan and Zisserman, 2015), ResNet50 (He et al., 2016), and Xception (Chollet, 2017). The selection of these architectures was based on findings from prior studies exploring DL for medical image analysis (Aggarwal et al., 2021; Anaya-Isaza et al., 2021). For each of the three CNNs, we omitted the final fully connected (FC) layer from the original architecture, initializing all other layers with weights derived from the respective networks trained on ImageNet (Figure 1D). ImageNet is an extensive open image dataset encompassing over 1 million diverse examples, spanning various objects and environments, from wildlife and livestock to vehicles, airplanes, and household items (Deng et al., 2009). This approach, termed Transfer Learning by Weiss et al. (2016), accelerates the training process by initializing network weights with values optimized for a comprehensive generic image dataset like ImageNet, rather than relying on random initialization.

All network architectures were extended with a global average pooling layer (Lin et al., 2014), followed by batch normalization, two FC hidden layers of size 128 and 64, and an output layer of size 1 (Figure 1E). The ReLU was used as an activation function for the hidden layers, whereas the sigmoid was adopted for the output layer. To prevent overfitting, dropout rates of 0.2, 0.1, and 0.05 were adopted for the average pooling layer and the 2 hidden layers, respectively. Additionally, weight regularization was imposed in the same extended layers. We opted to maintain a consistent overall structure when extending the pre-built architecture to ensure direct comparison across different architectures. For each CNN, the training process was divided into 2 stages: feature extraction and fine-tuning. In the feature extraction stage, we froze the original layers of the pre-built architectures to utilize previously learned features for our task. This process involved updating the weights of our extended layers for 50 epochs with a learning rate of 1 × 10^−2^. Subsequently, in the fine-tuning stage, all layers were unfrozen, and the networks were trained for an additional 100 epochs, considering a learning rate of 1 × 10^−4^. We adopted a batch size of 32 for all networks investigated. The Adam optimizer was used to update the FC weights during the feature extraction stage, while the SDG algorithm was adopted during the fine-tuning process. The described networks were implemented in Keras (Chollet et al., 2015) library available in Python, with Tensorflow (Abadi et al., 2016) as backend.

### Validation strategy and classification metrics

In practical applications, inferences are typically made at the cow level rather than on individual images. To evaluate the performance of the proposed approach, we employed a restricted cross-validation strategy to ensure that images from the same animal were not present simultaneously in the training, validation, and testing sets. We performed a 5-fold cross-validation by dividing the dataset into 5 folds. In each iteration, four folds (approximately 80% of the data) were used for training, while the remaining fold served as the independent testing set. Within each training iteration, the training set was further split: approximately 75% was used for model training, and the remaining 25% was designated as the validation set. The validation set was used to monitor classification performance and apply early stopping to save the best weights for the current fold. This process was repeated 5 times, with each fold serving as the testing set once. Inferences at the individual cow level were determined by averaging the network outputs across all valid frames extracted from the video recorded for each animal. The final inference at the individual cow level can be represented as ${\hat{p}}_{i}=\underset{t=1}{\overset{k_{i}}{\sum }}\;{\hat{p}}_{it}/k_{i}$, where ${\hat{p}}_{i}$ is the average probability of the *i*^*th*^ cow being pregnant, ${\hat{p}}_{it}$ is the network output of the sigmoid function for cow *i* at frame *t*, and $k_{i}$ is the number of valid frames extracted from the ultrasound video recorded for the *i*^*th*^ cow. The Youden’s index was used to select the optimal threshold point of ${\hat{p}}_{it}$ for each classifier (Fluss et al., 2005). We evaluated the classification performance of each architecture individually (VGG19, ResNet50, Xception) and, additionally, by averaging the output of the three CNNs tested (Combined). This averaging approach aimed to mitigate the strengths and weaknesses inherent in each architecture. The performance of the DL algorithms in distinguishing between pregnant and nonpregnant cows was assessed based on the values for accuracy, specificity, sensitivity, F1 score, and Matthew’s correlation coefficient (MCC) obtained in the testing sets. These metrics can be expressed as follows:


$$\begin{array}{l} accuracy\;\;\;=\;\frac{\left(TP+TN\right)}{\left(TP+TN+FP+FN\right)}, \end{array}$$

$$\begin{array}{l} specif\;icity=\;\frac{\left(TN\right)}{\left(TN+FP\right)}, \end{array}$$

$$\begin{array}{l} sensitivity=\;\frac{\left(TP\right)}{\left(TP+FN\right)}, \end{array}$$

$$\begin{array}{l} F1=\frac{TP}{TP+\frac{1}{\text{2}}(FP+FN)},\;and \end{array}$$

$$\begin{array}{l} MCC\;=\;\frac{TP\times TN-FP\times FN}{\sqrt{\left(TP+TN)(TP+FN)(TN+FP)(TN+FN\right)}}, \end{array}$$

where TP (True positives) represents the pregnant cows correctly classified, TN (True negatives) represents the nonpregnant cows correctly classified, FP (False positives) are the nonpregnant cows misclassified as pregnant, and FN (False Negatives) represent the pregnant cows misclassified as nonpregnant. Additionally, we compared the models based on the area under the Receiver Operating Characteristic (ROC) curve (area under the curve).

## Results

**Figure 2.**
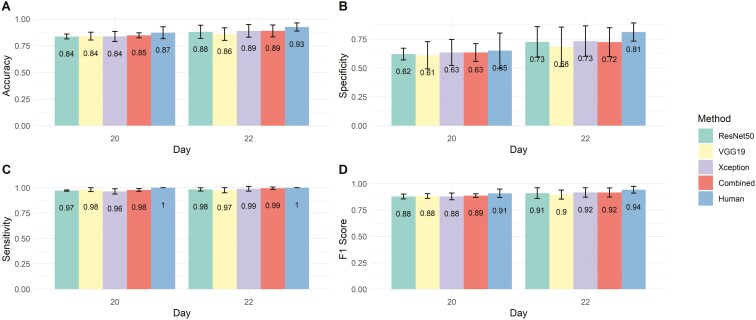
Bar plots of classification metrics for pregnancy assessment in beef cows based on color Doppler ultrasound data acquired 20 and 22 d after fixed-time artificial insemination. The comparison includes human-based evaluation and different convolutional neural network (CNN) architectures. Error bars represent the standard deviation of the classification metrics obtained for the CNNs across the testing sets. For human-based performance, error bars are included only for illustrative purposes. The CNN classification metrics were calculated using a standard threshold of 0.5 to classify a cow as pregnant.

**Figure 3.**
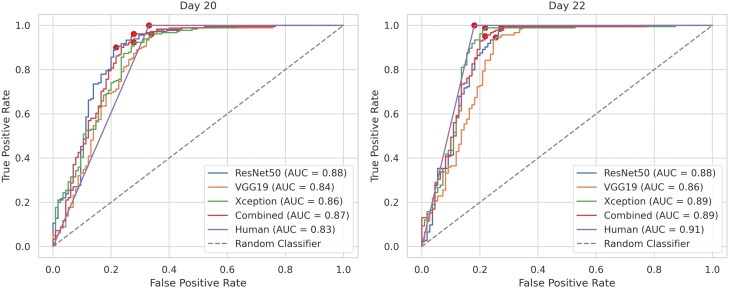
Receiver Operating Characteristic (ROC) curves and area under the curve (AUC) for pregnancy classification in beef cows, comparing human evaluation with different convolutional neural network (CNN) architectures. The pregnancy assessments were based on Doppler ultrasound data collected 20 and 22 d after fixed-time artificial insemination. Circles on the ROC curves indicate the optimal balance between sensitivity and specificity, determined using Youden’s Index.

Figure 2 shows the classification performance of pregnancy status in beef cows as determined by CD ultrasonography, assessed by trained individuals (*Human*) and through computer vision analysis using different CNN architectures. The classification performance improved from day 20 to day 22, with all methods achieving higher values for accuracy, sensitivity, specificity, and F1-score (Figure 2). Overall, the DL methods presented high accuracy for pregnancy classification, ranging between 0.84 and 0.85 for D20 and 0.86 and 0.89 for D22 (Figure 3). These values were slightly lower than the performance achieved by the human diagnostic, with accuracies of 0.87 for D20 and 0.93 for D22. A similar trend was observed for the other metrics, with the DL algorithms achieving similar, still slightly lower, sensitivity and F1 scores compared to the human-based pregnancy classification in both evaluation days (D20 and D22). The highest discrepancy between human and DL-based diagnostics was observed for specificity on day 22, with the lowest performance observed for the VGG19 architecture (0.68), compared to the human performance (0.81). All results for the DL algorithms reported in Figure 2 used the threshold value of 0.5 to classify a cow as pregnant. The ROC curves plotted in Figure 3 illustrate the average impact of different threshold values on the sensitivity and specificity tradeoff achieved by the various CNN architectures when classifying cows’ pregnancy status on days 20 and 22 after AI.

Figure 3 suggests that for certain threshold values, it is possible to increase specificity without significantly sacrificing sensitivity. According to Youden’s index, the average threshold values for optimal balance between sensitivity and specificity on day 20 were 0.79, 0.56, 0.64, and 0.7 for the ResNet50, VGG19, Xception, and Combined architectures, respectively. On day 22, the optimal tradeoff was found at threshold values of 0.53, 0.73, 0.61, and 0.73, following the same order. The average ROC curves indicate that DL algorithms could achieve slightly better specificity than human-based assessments on day 20 and comparable results on day 22. Moreover, the area under the ROC curves (area under the curve) shows that all DL methods performed substantially better than a random classifier. However, none of the DL architectures investigated achieved the 100% sensitivity observed for the veterinary-based pregnancy diagnosis (Figure 3).

**Figure 4.**
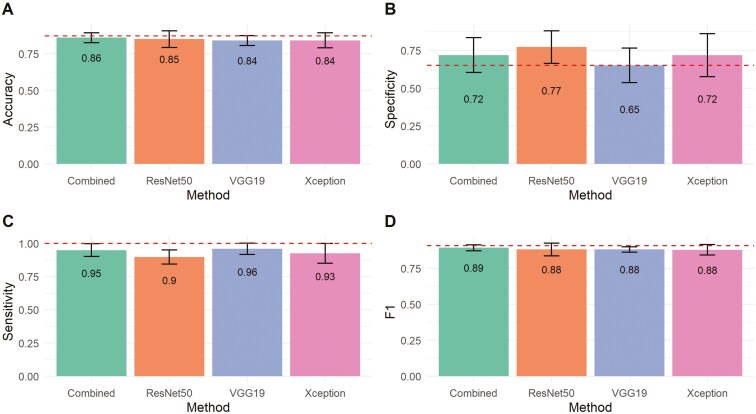
Bar plots of classification metrics for early pregnancy status prediction in beef cows based on different convolutional neural network (CNN) architectures trained with color Doppler ultrasound images recorded 20 d after fixed-time artificial insemination. The probability of a cow being pregnant was computed as the average of the CNN outputs across all valid frames extracted from the video recorded for each animal. The optimal probability threshold to classify cows as pregnant was defined using Youden’s Index. Error bars represent the standard deviation of the classification metrics obtained for the CNNs across the cross-validation. The dashed line represents the performance for human-based pregnancy classification in controlled conditions.

**Figure 5.**
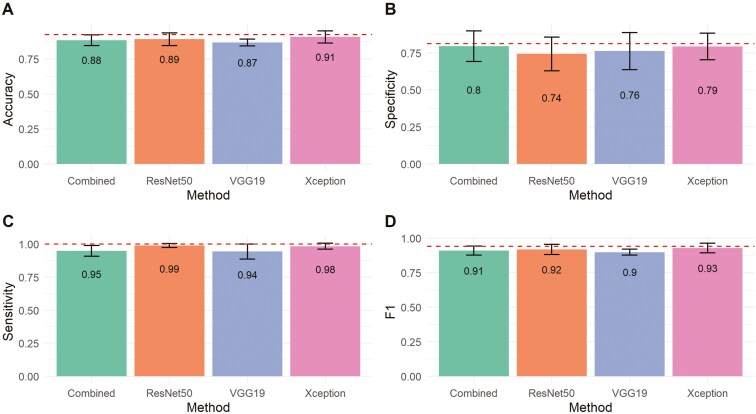
Bar plots of classification metrics for early pregnancy status prediction in beef cows based on different convolutional neural network (CNN) architectures trained with color Doppler ultrasound images recorded 22 d after fixed-time artificial insemination. The probability of a cow being pregnant was computed as the average of the CNN outputs across all valid frames extracted from the video recorded for each animal. The optimal probability threshold to classify cows as pregnant was defined using Youden’s Index. Error bars represent the standard deviation of the classification metrics obtained for the CNNs across the cross-validation. The dashed line represents the performance for human-based pregnancy classification in controlled conditions.

We further explored how thresholds selected according to Youden’s index across the cross-validation scheme influenced the classification metrics. Figures 4 and 5 show that, in most cases, the optimal threshold selection significantly increased specificity on both evaluation days compared to the standard threshold of 0.5 (Figure 2), without substantially reducing sensitivity. Additionally, accuracy and F1-score were maintained at the same or higher levels. For instance, with the optimal threshold, the specificity of the combined architecture on day 20 increased from 0.63 to 0.72, surpassing the specificity baseline achieved by the Human method (Figure 4), while the average sensitivity decreased slightly from 0.98 to 0.95. Figures 4 and 5 also indicate that the baseline accuracy and F1-score obtained by trained veterinarians fall within the variation expected for the tested CNN architectures, suggesting comparable performance between highly trained individuals and DL algorithms in classifying pregnancy in beef cows based on CD ultrasound imaging. Notably, the Combined architecture achieved the highest F1 score on day 20 (F1 = 0.89), while Xception performed best among the DL methods on day 22 (F1 = 0.93).

**Figure 6.**
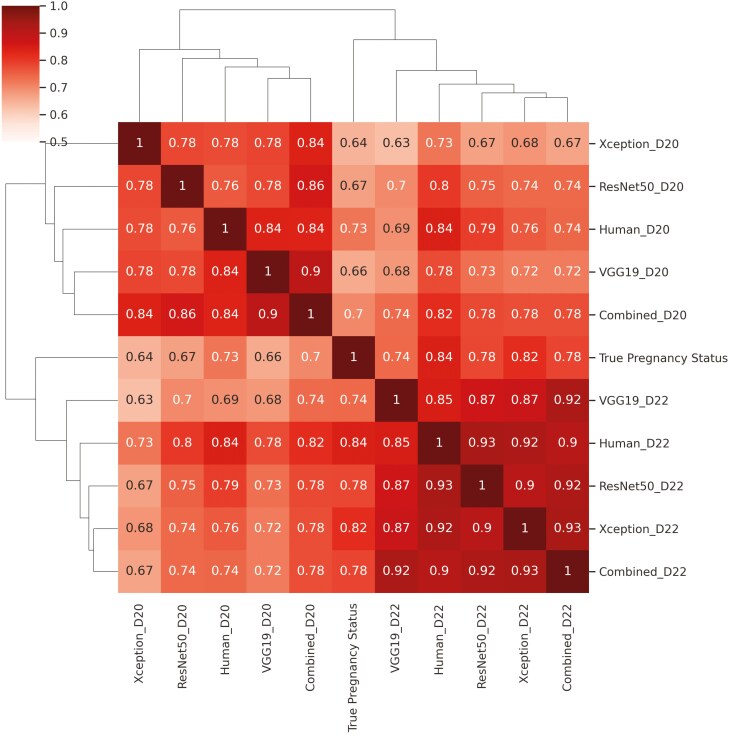
Mathew’s correlation coefficients among early pregnancy classification in beef cows predicted by different convolutional neural networks (CNN) architectures and highly skilled personnel (Human) based on Color Doppler ultrasonography recorded 20 (D20) and 22 (D22) d after fixed-time artificial insemination.

We evaluated the overall agreement among the different methods tested and with the final pregnancy status using Matthew’s Correlation Coefficient (MCC) estimated from the testing sets (Figure 6). The overall agreement among CNN methods ranged from 0.63 (Xception on day 20 with VGG19 on day 22) to 0.93 (ResNet50 and Combined, both on day 22), indicating moderate to high agreement among the classifiers. Notably, an MCC of up to 0.93 was observed between the Human and CVS-based pregnancy classifications on day 22. This finding reinforces that the proficiency of highly trained personnel in detecting pregnant and nonpregnant cows through Doppler ultrasound can be accurately replicated in real-time using DL methods. Additionally, the MCC values indicate strong agreement between both human-assessed pregnancy (up to 0.84) and computer vision classifications (up to 0.82) with the true pregnancy status (Figure 6).

**Figure 7.**
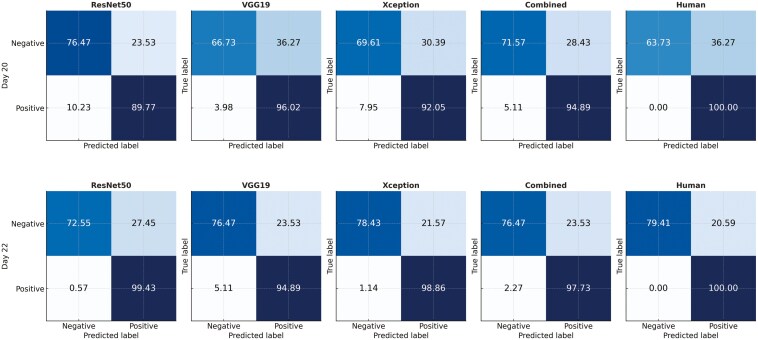
Confusion matrices obtained for early pregnancy classification in beef cows predicted by different convolutional neural networks (CNN) architectures and highly skilled personnel (Human) based on Color Doppler ultrasonography recorded 20 (D20) and 22 (D22) d after fixed-time artificial insemination.

Figure 7 presents the confusion matrices illustrating the performance of veterinarians and various computer vision methods in classifying cow pregnancy based on CD ultrasonography recorded 20 and 22 d after FTAI. On day 20, the false positive rate (FPR) for human-based evaluations was 36.27%, while the FPR for computer vision methods ranged from 23.53% (ResNet50) to 36.27% (VGG19). In contrast, the false negative rate (FNR) for CVS on the same day ranged from 3.98% to 10.23%, whereas human-based diagnoses showed no FN on either D20 or D22, as also indicated by the reported sensitivity values. A clear reduction in both FPR and FNR was observed for DL methods when comparing days 20 and 22 (Figure 7). The *Xception* architecture demonstrated the best performance balance on day 22, with a minimal FNR of 1.14% and an FPR of 21.57%, closely aligning with the errors observed in human evaluations on that day (Figure 7).

## Discussion

Early pregnancy detection allows beef cattle producers to promptly rebreed nonpregnant females after a failed insemination. This reduces calving intervals and increases the proportion of females that conceive early in the breeding season. Consequently, it leads to greater weaning weights (Funston et al., 2012) and improved pregnancy rates in subsequent breeding seasons (Cushman et al., 2013; Stevenson et al., 2015), enhancing productive and reproductive efficiency and maximizing economic gains for cow-calf operations (Lamb et al., 2016). CD ultrasonography has been proposed as a viable method for pregnancy diagnosis as early as day 20 of gestation. Recent literature indicates that human-based pregnancy diagnosis using CD ultrasound achieves accuracy values ranging from 87% to 93% when performed between days 20 and 22 of gestation in beef cattle (Fontes and Oosthuizen 2022). These findings align with the accuracy observed for human-based evaluations in our study. However, while CD ultrasonography allows for earlier pregnancy diagnosis compared to conventional methods, it relies on highly trained veterinarians for processing and interpretation. Additionally, the subjective nature of current blood perfusion assessment methods may exhibit inter- and intra-observer variability (Siqueira et al., 2013; Pugliesi et al., 2014). These limitations restrict its widespread adoption in commercial settings (Fontes and Oosthuizen 2022). To address these challenges, we explored the use of supervised DL algorithms for early, automated, and objective pregnancy classification based on CD images. To the best of our knowledge, this is the first study to combine these technologies for pregnancy classification in livestock animals. It is important to emphasize that our goal is not to replace veterinarians in pregnancy diagnosis but to leverage computer vision techniques as a fast and reliable method to assist these professionals with chute-side visual assessment of CD ultrasounds under commercial conditions. Appropriately trained CNN models could be embedded into ultrasound machines or in connected devices to receive the image streaming and infer a numerical probability of pregnancy in real-time, offering critical support to facilitate image interpretation, reduce misdiagnosis, and optimize the workflow of ultrasound analysis in farm conditions.

Our results indicate that the performance of DL algorithms is comparable to that of trained human experts, demonstrating that CVS can effectively support reproductive management decisions in commercial settings based on CD imaging. The accuracy of pregnancy classification using DL methods ranged from 0.84 to 0.86 on day 20 and from 0.88 to 0.91 on day 22 after FTAI, surpassing the previously reported accuracy of 0.79 for CNN-based classification of pregnancy status in cows using B-mode ultrasonography of ovarian structures recorded 30 d post-insemination (Andrade et al., 2023). Additionally, our approach achieved performance that is superior to or comparable with other algorithm-driven methods for automated pregnancy diagnosis in cows, such as fetus electrocardiogram and phonocardiogram signal processing (sensitivity = 0.876, specificity = 0.746; Gargiulo et al., 2012), infrared spectra combined with milk composition (AUC ranging from 0.607 to 0.645; Toledo-Alvarado et al., 2018), mid-infrared spectroscopy (accuracy = 0.88; Brand et al., 2021), and various biomarkers integrated with machine learning algorithms (accuracy ranging from 0.70 to 0.74; Ferraz et al., 2024).

Accurate pregnancy diagnosis can be performed manually with B-mode ultrasonography by detecting key structures such as the conceptus, embryo/fetal heartbeat, amniotic and chorioallantoic fluid, CL, and placental membranes (Beal et al., 1992). This method is highly accurate from around 30 d of gestation when conducted by skilled personnel (Beal et al., 1992; Y. Ribadu and Nakao, 1999). However, the accuracy of pregnancy detection with B-mode ultrasound rapidly decreases for gestational ages <28 d (Pieterse et al., 1990; Badtram et al., 1991; Nation et al., 2003; Romano et al., 2006). Previous studies have shown that CD imaging offers more detailed insights into CL function compared to conventional B-mode ultrasonography. CD imaging can reveal temporal changes in both CL morphometry and blood perfusion during early gestation (Herzog et al., 2010; Pugliesi et al., 2014). Moreover, as nonpregnant cows undergo luteolysis, the rate of decrease in circulating concentrations of progesterone is greater than the rate of decrease in CL area (Kastelic et al., 1990; Assey et al., 1993; Rocha et al., 2019), while decreases in luteal blood perfusion seem to occur before a structural regression of the CL can be noticed (Herzog et al., 2010; Rocha et al., 2019). This suggests that CD imaging offers more detailed features that can be leveraged by both trained personnel and DL algorithms to improve accuracy in detecting pregnancies at earlier stages compared to B-mode ultrasonography.

The values of accuracy, specificity, sensitivity, F1 (Fig. 4 and 5), and MCC (Fig. 6) indicate that the averaged inference from the 3 architectures (VGG19, ResNet50, and Xception) achieved the best overall performance on day 20, while the Xception architecture performed the best on day 22. Averaging is one of the simplest ensemble methods; it involves taking the average predictions from multiple base models. This method is particularly effective when individual models have diverse strengths and weaknesses. By adopting this approach, we aimed to explore the design differences in the DL architectures investigated. VGG19 has considerably more parameters but uses shallower architecture with standard convolutional layers, whereas ResNet50 and Xception employ deeper architectures with more sophisticated layers. ResNet50 uses residual connections to address issues like vanishing gradients, while Xception leverages depthwise separable convolutions for greater computational efficiency. On the other hand, the lower accuracy found for VGG19 on day 22 indicates that its very large number of parameters may have led to overfitting or inefficiencies in feature extraction, thereby hindering its classification performance compared to the other architectures.

Overall, DL methods exhibited fewer false positive errors than the human-based evaluation on day 20, while the FPRs for the tested CNN architectures were slightly higher than those achieved by trained individuals on day 22. The factors contributing to FPRs in pregnancy diagnosis using luteal CD ultrasonography—whether assessed by human or computer vision methods—are not yet fully understood. Research indicates that pregnancy loss between days 20 and 29, increased luteal lifespan, and lack of ovulation synchrony are potential drivers of false positives (Dalmaso de Melo et al., 2020; Holton et al., 2022a,b). This suggests that the suboptimal specificity observed with both human and DL approaches may reflect a biological limitation for the use of CD to determine pregnancy status. Additionally, the comparable results between CVS and human methods suggest that this biological relationship is predominantly captured by observable luteal features such as size and blood perfusion. Features like echogenicity and complex hidden relationships, which DL algorithms are expected to better explore, appear to play a minimal role in this context.

For commercial deployment, early pregnancy detection methods must achieve 100% sensitivity. This is crucial because incorrectly diagnosing pregnant cows as nonpregnant (false negatives) and subjecting them to a second service could increase the risk of losing the initial pregnancy. Human-based CD ultrasonography, as performed in this study, achieves 100% sensitivity with specificity ranging between 72% and 83% (Dalmaso de Melo et al., 2020; Holton et al., 2022a,b). In comparison, the computer vision methods showed specificities within this range but presented FNR ranging between 3.98% and 10.23% for day 20 and between 0.57% and 5.11% for day 22 (Figure 7). These results impose limitations on the commercial application of the methods investigated in this study, highlighting the need for further research to address these challenges. The limited number of videos available may have affected the model’s ability to generalize across animals. Since multiple frames were extracted from each video, this introduced temporal dependencies among the images, thereby reducing the diversity of unique representations that CNN could learn. Increasing the number of animals in the training dataset could help mitigate this issue and contribute to a lower FNR in future applications. On the other hand, it is important to note that the human interpretation of CD videos in this study was conducted under controlled conditions rather than in practical chute-side settings. Consequently, the reported results may represent an upper bound for what is expected for human-based diagnosis in real farm conditions.

In this study, inferences for individual frames within videos were performed independently and then averaged to predict cow-level pregnancy status. Consequently, our approach focused solely on spatial features, neglecting temporal dependencies between frames. This simplification aimed to facilitate data augmentation by allowing multiple frames to be extracted from each video. However, this approach limits our ability to explore how temporal dynamics might influence predictions. Incorporating 3D CNNs, hybrid LSTM-CNN architectures, or other methods that capture both spatial and temporal features could potentially enhance accuracy compared to the regular CNN architectures typically used in medical ultrasound image analysis (Zhang et al., 2022; Zhao et al., 2023). Nonetheless, implementing such networks in our dataset could be challenging due to the relatively small sample size available, which could lead to overfitting or inadequate training. Additionally, the computational demands of video-based classification methods present a significant barrier to real-time inference, which is crucial for our practical monitoring solutions.

Despite current limitations, the DL methods investigated in this study have the potential to provide real-time support for human-based pregnancy diagnosis in commercial applications, particularly for veterinarians who may lack the specialized reproductive physiology training of scientists validating these methodologies for CD ultrasound evaluation. The proposed approach could contribute to mitigating subjectivity reported in CD evaluations (Pugliesi et al., 2014; Fontes and Oosthuizen 2022). Additionally, while this study focused on CNNs for direct pregnancy classification, other computer vision techniques could also contribute to optimizing the workflow of ultrasound analysis in farm settings. For example, segmentation or object detection algorithms could be used to identify regions of interest in ultrasound images, facilitating the calculation of useful metrics such as luteal area, diameter, and blood perfusion. Moreover, CVS applications in female reproductive management are not limited to pregnancy diagnosis; to name a few examples, computer vision could also be valuable for fetal aging, monitoring ovarian health, assessing follicular development, evaluating responses to fertility treatments, and tracking embryo and placenta developmental kinetics.

In conclusion, DL algorithms can successfully predict pregnancy status using CD ultrasonography earlier than traditional industry methods, showing comparable performance to diagnoses conducted by trained individuals in controlled environments. Our initial findings suggest that integrating CD ultrasound data with DL algorithms offers a promising solution to support veterinarians and farmers in automated and early pregnancy diagnosis. Nonetheless, additional research is necessary to enhance the sensitivity of this approach before it can be adopted commercially.

## Abbreviations:

AI: artificial insemination
AUC: Area under curve
B-mode: Brightness mode
CD: color Doppler
CL: corpus luteum
CNN: convolutional neural network
CVS: computer vision systems
DL: deep learning
D20: day 20 after fixed-time artificial insemination
D22: day 22 after fixed-time artificial insemination
FC: fully connected
FN: false negatives
FNR: false negative rate
FP: false positives
FPR: false positive rate
FTAI: fixed-time artificial insemination
Human: human-based pregnancy diagnosis
MCC: Matthew’s correlation coefficient
ROC: receiver operating characteristic
TN: true negatives
TP: true positives
US: ultrasound
